# Traumatic Tetanus Successfully Treated by Friction with Chloroform

**Published:** 1851-11

**Authors:** 

**Affiliations:** Physician to the District Hospital at La Fleche (Barthe.)


*Traumatic Tetanus successfully treated by Friction with Chloroform.* By Dr. Morisseau, Physician to the District Hospital at La Fleche (Barthe.)—The patient was about forty years old, a man of good constitution. Eight days before admission, he had slightly wounded himself on the front of his leg, while at work with his pick-axe. The trifling wound had healed by the fifth day. On the sixth day tetanic symptoms manifested themselves.

The patient was at first treated at home, but the disease advancing, he was removed to the hospital. When first seen by Dr. Morisseau, the tetanus was fully developed; there was persistent contraction of the muscles of the jaw, the chest, abdomen, and back; the body was bent double every hour; deglutition impossible. The pulse small and slow; nothing particular was observable of other functions.

Friction was performed with four grammes (one drachm) of chloroform. This quantity was repeated three times during the day. The patient was placed in an acidulated vapor bath.

On the following day a considerable improvement had taken place. The patient had perspired freely; he had slept well which he had not done before, since the commencement of the attack. He had swallowed a few spoonsful of liquid; the muscles were less rigid; convulsions shorter and less frequent.

The dose of chloroform was increased to twenty grammes, three times a day. This treatment was continued for five days with the best results; all the symptoms had disappeared on the sixth day. A sense of lassitude and debility alone remained, and these soon disappeared under suitable diet.

The editor of the *L' Union Médicale* adds to the above the following taken from the *Gazetta Medica Lombarda*: A laborer, twenty-eight years of age, was seized with tetanus two days after having lain on damp ground whilst he was in a perspiration. The patient was immediately subjected to the treatment usually adopted there, and which consists in frequent venesection. He was bled eight times in four days, once to the amount of twenty ounces; about a hundred leeches were applied to the parts that were painful, and other ordinary means were adopted. On the sixth day the patient was in a very serious state, when M. Tibaldi had recourse to frictions with ether in order to calm the muscular contractions. Two frictions were practised on the loins; venesection and half a grain of acetate of morphia was given. Whenever friction was applied, the action of the muscles was moderated. On the following day another bleeding to ten ounces, and frictions with ether on the neck and back. On the third day of this treatment the patient could sit upright. In two or three days more the patient was convalescent.—*Lon. Med. Gaz.*

